# Antibody responses to *Borrelia burgdorferi* detected by western blot vary geographically in Canada

**DOI:** 10.1371/journal.pone.0171731

**Published:** 2017-02-09

**Authors:** Nicholas H. Ogden, Julie Arsenault, Todd F. Hatchette, Samir Mechai, L. Robbin Lindsay

**Affiliations:** 1 National Microbiology Laboratory, Public Health Agency of Canada, Saint-Hyacinthe, Québec, Canada; 2 Groupe de recherche en épidémiologie des zoonoses et santé publique (GREZOSP), Saint-Hyacinthe, Québec, Canada; 3 Département de pathologie et microbiologie, Faculté de médecine vétérinaire, Université de Montréal, Saint-Hyacinthe, Québec, Canada; 4 Department of Pathology and Laboratory Medicine, Nova Scotia Health Authority, Halifax, Nova Scotia, Canada; 5 Department of Pathology, Dalhousie University, Halifax, Nova Scotia, Canada; 6 National Microbiology Laboratory, Public Health Agency of Canada, Winnipeg, Manitoba, Canada; Umeå University, SWEDEN

## Abstract

Lyme disease is emerging in eastern and central Canada, and most cases are diagnosed using the two-tier serological test (Enzyme Immuno Assay [EIA] followed by Western blot [WB]). Simplification of this algorithm would be advantageous unless it impacts test performance. In this study, accuracy of individual proteins of the IgG WB algorithm in predicting the overall test result in samples from Canadians was assessed. Because *Borrelia burgdorferi* strains vary geographically in Canada, geographic variations in serological responses were also explored. Metrics of relative sensitivity, specificity and the kappa statistic measure of concordance were used to assess the capacity of responses to individual proteins to predict the overall IgG WB result of 2524 EIA (C6)-positive samples from across Canada. Geographic and interannual variations in proportions of samples testing positive were explored by logistic regression. No one protein was highly concordant with the IgG WB result. Significant variations were found amongst years and geographic regions in the prevalence of samples testing positive using the overall IgG WB algorithm, and for individual proteins of the algorithm. In most cases the prevalence of samples testing positive were highest in Nova Scotia, and lower in samples from Manitoba westwards. These findings suggest that the current two tier test may not be simplified and continued use of the current two-tier test method and interpretation is recommended. Geographic and interannual variations in the prevalence of samples testing positive may be consistent with *B*. *burgdorferi* strain variation in Canada, and further studies are needed to explore this.

## Introduction

In North America Lyme disease is caused by the tick-borne bacterium *Borrelia burgdorferi* sensu stricto, (hereafter shortened to *B*. *burgdorferi*). The most frequently used laboratory method for Lyme disease diagnosis is serology using the two-tier (Enzyme Immuno Assay [EIA] followed by Western blot [WB]) algorithm. This algorithm, when using specific interpretation criteria, is the method recommended by infectious disease experts [1, 2] and public health organisations such as US Centres for Disease Control & Prevention (CDC) and the Public Health Agency of Canada. Sensitivity of this two-tier algorithm has been questioned by some patient groups and medical practitioners [3, 4], but alternative interpretation criteria used by some private laboratories in the US have high (>50%) false positive rates [5] and patients may be being falsely diagnosed as having “chronic Lyme disease” [6]. Using CDC Western blot interpretation criteria, the two-tier test has some limitations to specificity as false-positivity has been detected associated with cross-reactivity to other infections [7], and in early Lyme disease specific antibody levels are low so test sensitivity is low (ca 30%) [1]. However for early Lyme disease cases with typical cutaneous Erythema migrans (EM) lesions [8, 9], sero-diagnosis is not needed. In early disseminated Lyme disease some patients may also test negative, although with progression of infection to late disseminated Lyme disease, test sensitivity increases [1]. For these reasons research on novel methods of laboratory diagnosis is underway [10], and research has also aimed to see if the somewhat complicated two-tier test algorithm can be simplified without significantly impacting on test performance [11].

Studies suggest that the infecting strain of *B*. *burgdorferi* can influence the sensitivity of the two-tier test, particularly in early Lyme disease in humans [12], but also in later infections in experimental animals [13]. These possible effects of strain variation on sero-diagnosis may be particularly pertinent to Canada as in some locations a significant proportion of the strains are different to those found in the US [14]. It may be prudent, therefore, to investigate whether there is any evidence for this geographic pattern of strains to have impact on serological test performance in Canada.

Using sera submitted to the national reference laboratory for Lyme disease testing the objectives of this study were to determine if any protein of the Western blot was particularly predictive of the overall Western blot result in Canada, and to examine WB banding patterns for evidence of geographic variations in the serologic response to proteins in the CDC WB algorithm that could, theoretically, be associated with observed geographical variations in occurrence of different strains of *B*. *burgdorferi* [14].

## Materials & methods

### Samples used in the study and WB analysis

This was a retrospective analysis of data generated by serologic testing for Lyme disease conducted, using an ISO 17025 accredited two tier algorithm based on CDC recommendations, at the National Microbiology Laboratory (NML, Winnipeg, Manitoba) on diagnostic specimens submitted from 2011 to end of April 2015 by provincial laboratories in Canada. The study used samples submitted in the normal course of laboratory diagnosis for Lyme disease in Canada that, apart from the year of submission and province of origin, contained no patient data. Internal review within the Public Health Agency of Canada concluded that for statistical analysis of existing results from such samples, ethical review is not necessary. Provincial laboratories conduct a screening ELISA test and then send ELISA-positive or equivocal samples to NML Winnipeg for confirmatory testing by the two-tier method. Thus, with the exceptions of Ontario and British Columbia, where the provincial laboratories conduct both ELISA and Western blot testing (but occasionally send samples to NML for confirmatory testing), the test data analysed here represent all the Lyme disease tests conducted in Canada during the study period. At NML, the submitted samples were subject to the full two tier test. They were tested with a screening C6 ELISA (Immunetics^™^), the first test in the two-tier algorithm [3]. Specimens testing positive or equivocal were then subject to WB analysis (the second test of the two-tier algorithm) using commercially-available kits (Anti-*Borrelia burgdorferi* US EUROLINE-WB (IgG), Euroimmun^™^) following manufacturer’s instructions. Consistent with CDC criteria [3], an IgG WB was considered positive if reactions to 5 of 10 bands were detected (corresponding to the proteins p18/21, p25. p28, p30, p39, p41, p45, p58, p66 and p83/93). Only responses to IgG antibodies were considered in the analysis. We recorded the overall WB result, and whether or not there was a positive response to each individual protein. All WBs were read and interpreted using an automated scanner and reader (Euroimmun AG, Luebeck, Germany). Individual WB membrane strips were scanned onto a protocol sheet or photographed directly and proprietary software (EurolineScan, http://www.euroimmun.us/our-products/automation/eurolinescan) was used to score individual bands. The EurolineScan identified the bands and measured the intensity of each. The relative intensity of each band was automatically provided and for the purposes of this study scored as absent or reactive/positive. The data and test results are presented in supporting information (S1 File).

### Statistical analysis

#### Concordance of IgG responses to individual proteins with the IgG WB result

We explored the concordance of each band of the CDC algorithm with the overall WB test result to see if responses to particular proteins had high concordance with the overall two-tier test result (based on IgG WB results). The metrics for comparison were relative specificity, relative sensitivity and the kappa statistic measure of concordance of each protein compared with the overall WB test result, which were calculated in Stata SE 11.0 (College Station, Tx, USA).

#### Geographical variations in responses

Evidence for geographic variations in the presence of responses to different proteins possibly attributable to geographic variations in strains was explored using multiple methods, including those aimed at reducing the possibility of bias.

First, the overall IgG WB test result was the outcome in logistic regression in which province of sample origin and year of test were categorical explanatory variables. The provinces were aggregated to account for known geographic variations in the environmental risk from Lyme disease. Risk from *B*. *burgdorferi* transmitted in established *Ixodes scapularis* tick vector populations occurs in Nova Scotia (NS), Quebec (QC), Ontario (ON) and Manitoba (MB), is thought to be absent from Newfoundland & Labrador (NFL), Prince Edward Island (PEI), Alberta (AB) and Saskatchewan (SK) and is uncommon in New Brunswick (NB) [15, 16]. Environmental risk from *B*. *burgdorferi* transmitted in established *Ixodes pacificus* tick vector populations occurs in southern British Columbia (BC) but here risk of acquiring Lyme disease where *I*. *pacificus* is the vector is much lower than where *I*. *scapularis* is the vector because of a range of ecological factors [17]. Consequently the provinces were grouped into five groups (or individual provinces) as follows: group 1 NS, group 2 NFL + PEI + NB, group 3 QC + ON, group 4 MB, group 5 AB + SK + BC. It is possible that samples submitted from provinces where there are no known tick vector populations at present (NFL, PEI, AB and SK) were predominantly from travellers, so this analysis was repeated using only data from provinces in eastern and central Canada where *I*. *scapularis* populations are known to be established, i.e. NS, NB, QC+ON and MB.

Second, correspondence analysis was conducted to identify any clusters of individual WB algorithm proteins that may be particularly commonly associated with certain province groups, and should therefore be analysed as distinct groups. Multiple correspondence analysis was performed using SPSS 17 version program (SPSS, Inc, Chicago, Illinois, USA) to identify relationships amongst individual proteins and province groups, while account for year of sampling. The outcomes of analysis were explored by visual inspection for spatial co-location of proteins and provinces in a biplot map, with the strength of any identified associations being assessed using Cronbach's alpha coefficient [18], for which a value > 0.5 suggests significant associations [19].

Third, logistic regression models were constructed with each protein in the WB algorithm as the outcome variable, and the province group of origin and year as categorical variables. The IgG WB test result was also included as an explanatory variable. Therefore any significant variations amongst provinces in the likelihood of positive reaction to a protein would be variations over and above those associated with the overall test result. This method was chosen because variations in the likelihood that a sample tested positive amongst provinces could be associated with geographic variations in strains, but could also be due to differences amongst provinces in the selection of patients and samples for testing that we are not aware of. This analysis was also repeated, first using data only from NS, NB, QC+ON and MB (i.e. excluding locations without known *I*. *scapularis* populations so reducing the likelihood that positive samples are false positive), and then using data only from WB-positive samples from these province groups further reducing the possibility of bias due to inter-provincial differences in reasons for submitting samples for testing.

Logistic regression analyses were also repeated excluding data from Ontario (from where samples were submitted for confirmatory testing for unknown reasons rather than in the normal course of Lyme disease diagnosis) and from locations without known *I*. *scapularis* populations.

Fourth, variations amongst provinces and years in the number of bands in IgG WB-positive and —negative samples separately were also explored by ordered logistic regression with again province group and year as explanatory variables. All statistical analyses were conducted in State SE version 11.0 (College Station, Tx, USA).

## Results

### Concordance of IgG responses to individual proteins with the IgG WB result

**Fig 1:**
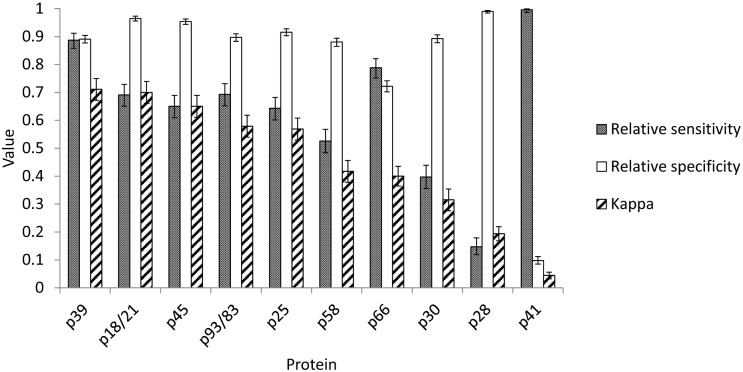
Values for the kappa statistic, and relative sensitivity and relative specificity of responses to individual proteins in predicting the IgG Western blot result. The proteins are ordered on the x-axis with decreasing kappa statistic values from left to right. 95% confidence intervals for relative sensitivity, specificity and the kappa statistic are shown.

Results were available from 2524 C6-positive/equivocal samples (9 from BC, 464 from AB, 29 from SK, 696 from MB, 17 from ON, 632 from QC, 183 from NB, 4 from PEI, 476 from NS and 14 from NFL). Responses to individual proteins varied considerably in their concordance with the overall WB result as measured by their kappa statistic values (Fig 1 and Table A in S2 File). Using standard criteria [20] none of the proteins had ‘almost perfect’ agreement (kappa > 0.8) with the IgG WB results, three proteins (p18/21, p39 and p45) had ‘substantial’ agreement (kappa = 0.61–0.8), three (p25, p58, p93/83) had ‘moderate’ agreement (kappa = 0.41–0.6), two (p30, p66) had ‘fair’ agreement (kappa = 0.21–0.40) and two (p28, p41) had ‘slight’ agreement (kappa = 0.01–0.20). The low kappa values for p28 and p41 were due, respectively, to low relative sensitivity and low relative specificity (Fig 1 and Table A in S2 File).

### Geographical and interannual variations in responses

**Fig 2:**
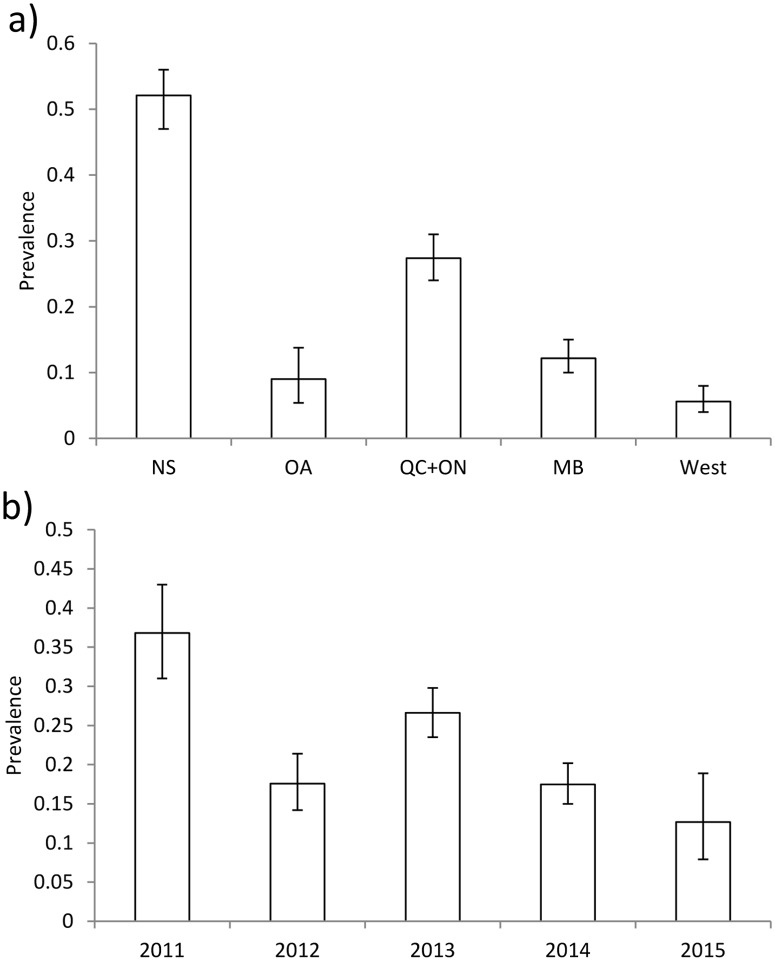
Variations in the prevalence (with 95% confidence intervals) of C6-positive/equivocal samples that tested positive using the IgG Western blot algorithm by groups of provinces (graph a) and by year (graph b). NS = Nova Scotia, OA = other Atlantic provinces (Newfoundland & Labrador, Prince Edward Island, and New Brunswick), QC = Quebec, ON = Ontario, MB = Manitoba, West = Saskatchewan, Alberta, and British Columbia.

There were geographical and interannual variations in the proportions of C6-positive/equivocal samples that tested positive according to the IgG WB algorithm. In general, the proportion of samples testing IgG WB positive was higher for provinces with the highest known environmental risk (NS, QC, ON and MB) than those with low environmental risk (NFL, NB, PEI, AB, SK and BC) (Table 1, Fig 2a). There were also differences amongst years with the proportion of samples testing IgG WB positive being highest in 2011 and 2013 (Fig 2b). Full model parameters are shown in Table B in S2 File. The results were similar when using only data from provinces where *I*. *scapularis* populations are known to be established (Table C in S2 File) and when excluding data from Ontario (Table D in S2 File).

**Table 1:** The numbers of C6-positive/equivocal samples tested, and the number (%) positive by the IgG Western blot algorithm, from each province and province group by year.

	Group 1	Group 2			Group 3		Group 4	Group 5			
Year	NS	NFL	PEI	NB	QC	ON	MB	SK	AB	BC	Total
2011	50/82 (61.0)	0/1 (0)	0/2 (0)	2/31 (6.5)	21/56 (37.5)	3/9 (33.3)	13/58 (22.4)	0/2 (0)	5/14 (35.7)	1/3 (33.3)	95/258 (36.8)
2012	39/77 (50.6)	0/5 (0)	0/0	3/38 (7.9)	20/90 (22.2)	0/7 (0)	12/146 (8.2)	1/12 (8.3)	6/83 (7.2)	0/2 (0)	81/460 (17.6)
2013	99/189 (52.4)	0/2 (0)	0/0	5/38 (13.1)	74/225 (32.9)	0/0	26/195 (13.3)	1/5 (20.0)	7/141 (5.0)	0/3 (0)	212/798 (26.6)
2014	52/115 (45.2)	0/5 (0)	0/2 (0)	5/61 (8.2)	54/230 (23.5)	0/1 (0)	31/256 (12.1)	0/9 (0)	7/170 (4.1)	0/1 (0)	149/850 (17.6)
2015	8/13 (61.5)	0/1 (0)	0/0	3/15 (20.0)	6/31 (19.3)	0/0	3/41 (7.3)	0/1 (0)	0/56 (0)	0/0	20/158 (12.7)
Total	248/476 (52.1)	0/14 (0)	0/4 (0)	18/183 (9.8)	175/632 (27.7)	3/17 (17.6)	85/696 (12.2)	2/29 (6.9)	25/464 (5.4)	1/9 (11.1)	557/2524 (22.1)

Abbreviations: NS = Nova Scotia, NFL = Newfoundland & Labrador, PEI = Prince Edward Island, NB = New Brunswick, QC = Quebec, ON = Ontario, MB = Manitoba, SK = Saskatchewan, AB = Alberta, BC = British Columbia

Correspondence analysis showed no evidence of clusters of proteins that were significantly associated with individual province groups on inspection of the resulting biplot and Cronbach’s alpha was less than 0.4 for both dimensions of all the analysis. Consequently responses to individual proteins were explored in separate logistic regression models.

**Fig 3:**
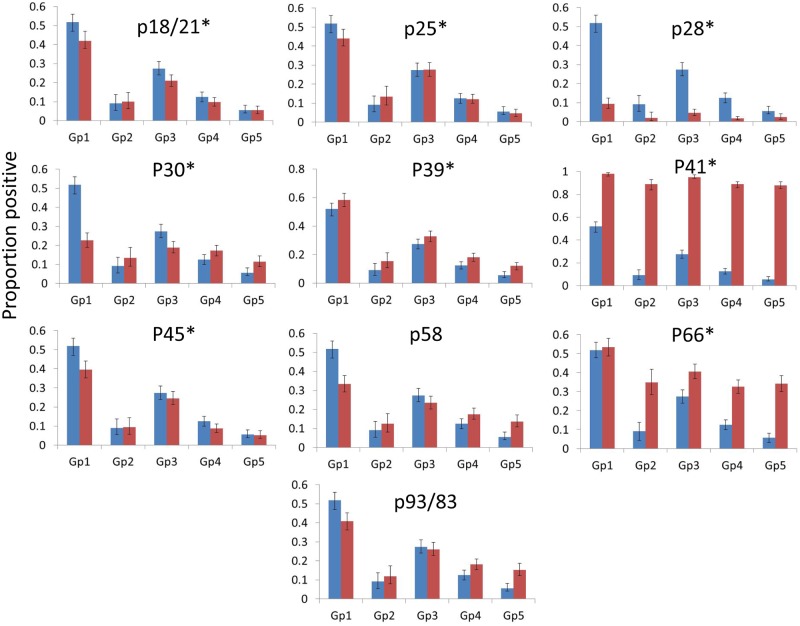
Variations amongst groups of provinces in the proportions of C6-positive/equivocal sera reactive to each protein of the IgG Western blot algorithm (red bars). For comparison, in each graph the proportions of C6-positive/equivocal sera that were positive for the IgG Western blot algorithm in the different groups of provinces are shown as blue bars. Asterisks indicate that these variations were significant accounting for interannual variations and either without or with accounting for the variations in overall positivity to the IgG Western blot result. Exact binomial 95% confidence intervals are shown. Gp = province group. Gp1 = Nova Scotia, Gp2 = Other Atlantic Provinces (Newfoundland & Labrador, Prince Edward Island and New Brunswick), Gp3 = Quebec and Ontario, Gp4 = Manitoba, Gp5 = Provinces west of Manitoba (Saskatchewan, Alberta and British Columbia).

Accounting for geographic and interannual variations in overall IgG WB results, there were additional significant variations amongst provinces and years in the proportions of C6-positive/equivocal sera that reacted to some of the individual proteins in the two-tier test algorithm (Table E in S2 File, Fig 3). For p18/21, p25, p39, p41 and p45, the proportions of samples positive to individual proteins were generally lower in samples from the west (samples from MB westward with lowest prevalence in samples from SK west) than in the east (samples from ON eastward with highest prevalence in samples from NS). The proportion of samples with responses to p28 was significantly lower in samples from MB compared to SK west, but similar in other provinces. In contrast, the prevalence of responses to p30 was higher in samples from MB compared to all other provinces, and similar in other provinces, and the prevalence of responses to p66 was higher in samples from SK west and similar in other provinces. There were variations amongst years in prevalence of samples positive to individual proteins that varied from the overall pattern of interannual variations in prevalence of samples positive in the IgG WB algorithm (Table E S2 File). The interannual variations in responses to individual proteins were very variable from one protein to another (Table E S2 File).

When using samples only from provinces where *I*. *scapularis* populations are established, and accounting for the overall IgG WB result, variations in responses to individual proteins amongst province groups remained significant and qualitatively similar to when all data were used in analyses (Table F in S2 File). Differences amongst years in the prevalence of responses to individual proteins were also similar when using this reduced dataset although significant differences amongst years in responses to p58 were also found (Table F in S2 File). When also excluding data from Ontario, the results of analyses were almost unchanged (Table G in S2 File). When using only data from IgG WB-positive samples from provinces where *I*. *scapularis* populations are known to be established, significant differences amongst provinces and years were found for p18/21 and p28, and amongst years for p25 (Table H in S2 File).

**Fig 4:**
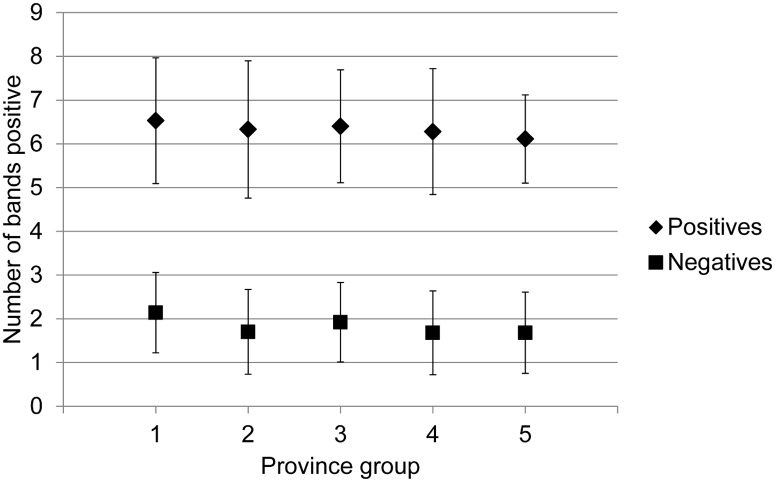
The mean (+/- SE) number of bands positive in IgG Western blot positive and negative samples for each of the different provinces or groups of provinces from which the samples came. Province groups are 1 = Nova Scotia, 2 = Other Atlantic Provinces (Newfoundland & Labrador, Prince Edward Island and New Brunswick), 3 = Quebec and Ontario, 4 = Manitoba, 5 = Provinces west of Manitoba (Saskatchewan, Alberta and British Columbia).

In ordered logistic regression analysis the geographic locations were significantly associated with the numbers of bands in IgG WB-negative samples, in a pattern similar to the IgG WB result and individual protein results, i.e. the number of positive bands in negative samples was more likely to be higher in NS, QC and ON, than in NB, NFL, PEI, MB, AB, SK and BC (Fig 4, Table I in S2 File). Lower numbers of bands were positive in 2014 and 2015 compared to earlier years (Table I in S2 File). This pattern was similar for IgG WB-positive samples (Fig 4) but was not significant.

## Discussion

In this study we used C6-positive/equivocal samples collected from patients across Canada, tested by a consistent method for all samples, to assess whether there was any evidence i) to support the use of fewer proteins in the WB algorithm when applying the test to Canadians, and ii) for geographic variations in the test performance in Canada that could be associated with geographic variations in the occurrence of strains of *B*. *burgdorferi* in wild animals and ticks to which Canadians are exposed. The geographic coverage of samples was as expected accounting for geographic variations in human population densities and environmental risk [9]. A low number of samples (17) were from Ontario even though this is the Canadian province with the highest number of Lyme disease cases [9], but this province is mostly autonomous in Lyme disease laboratory diagnostic testing.

We found that no one protein was particularly concordant with the overall WB result. All bands had different relative sensitivity and relative specificity characteristics, but those that showed higher concordance with the WB result (p18/21, p25 and p39) were also those that showed geographic variation in frequencies of positive results. The correspondence analysis revealed no evidence of clusters of proteins in the WB algorithm that had consistent patterns of positivity amongst the different province groups. Therefore, there was no evidence to support selection of different groups of protein bands for interpretation of the WB in different geographic regions. The p41 (flagellin) proteins had particularly low concordance due to low relative specificity which is consistent with the known cross-reactivity with this protein with similar proteins in other bacteria (e.g. [21, 22]). Overall our findings here do not support a simplification of the proteins used in the WB algorithm, and suggest that the use of the current WB algorithm components and interpretation should continue.

The proportion of C6-positive/equivocal tests that tested positive by WB was much lower than in reports from the US [11]. Given that the NML conducts stringent quality control including validation of test performance using known positive and negative panels of test samples provided by the US CDC and annual participation in external proficiency testing, it is unlikely that this finding represents different test sensitivity due to different testing modalities at NML. Some samples that were negative by IgG WB were positive by the IgM WB, but the IgM WB data were not investigated here as it is unknown whether these were true positive early Lyme disease (30 days or less since infection [3]) or false positive (> 30 days since infection) tests. However, even including these, only 34% of C6-positive/equivocal sera were positive by either IgM or IgG WB. It is possible that submission of samples for testing from patients with early Lyme disease, associated with poor physician awareness in Canada [23] may have contributed to low prevalence of WB-positive samples. However the precise reasons for the difference between the US and Canada in this regard remain to be investigated. Were C6 test sensitivity to be different in Canada, that would apply to all test results and would not explain geographic variations described in the following, and different patterns of submissions of samples in different provinces as a source of bias was accounted for in testing WB-positive samples only.

There was a geographic pattern in the proportions of C6-positive/equivocal samples that were positive by WB. This could suggest that positive C6 test results are more likely false positive in those provinces where there is low risk (British Columbia) or almost no risk (Saskatchewan and Alberta) from Lyme disease. However there may be other reasons why the prevalence of IgG WB-positive samples varied amongst provinces including different rates of submission of samples from early Lyme disease cases and submission of samples from travellers. Accounting for geographic variation in the overall WB result, which could have been due to factors other than environmental risk from Lyme disease, there was variation amongst provinces in the proportions of samples reactive to individual proteins. Mostly this variation comprised samples submitted from Manitoba and provinces west being less likely to be positive, and there was some evidence that this affected both positive and negative samples. In two cases however (p30 and p66), reactive samples were more common in samples from Manitoba and provinces further west (respectively) than elsewhere. There were similar findings when samples submitted from provinces with no known *I*. *scapularis* populations and samples from Ontario were removed from the analysis, and for p18/21 and p28 these findings remained significant when only data on IgG WB-positive samples from *I*. *scapularis*-endemic provinces were included in the analyses. By including the overall WB result in the models for individual proteins, restricting data used in analyses to those submitted from provinces with known *I*. *scapularis* populations, and by excluding data from Ontario, which may not have been submitted for the same reason as samples in other provinces with *I*. *scapularis* populations, we minimised the possibility of inter-provincial biases causing the observed geographic patterns. Therefore these findings may be consistent with geographic variation in the occurrence of strains having an impact on WB positivity. In the Maritimes, Quebec and eastern Ontario, most strains are the same as, or recent descendants of, strains in the northeastern US, which include B31 and other strains used in the WB preparations [24]. In Northwestern Ontario and Manitoba, the strains are more frequently unique, albeit descendants of strains originating in the Midwestern US [14], and we speculate that such strains may have a different capacity to elicit immune responses detectable using northeastern strains as antigen in the WB. A greater diversity of infecting *B*. *burgdorferi* strains in patients from the upper Midwestern USA compared to those from the northeastern USA has also been previously detected [25].

Further study may of course reveal alternative explanations for the observed geographic variations. In Europe, geographic variation in background immunity of the human population has been speculated as a cause of geographic variations in serological responses [26]. This possibility cannot be ruled out, but it seems an unlikely explanation here as i) Lyme disease emergence is at an early stage in eastern and central Canada so the human population exposed to date is small (only 2095 cases were reported nationally from 2009 to beginning of 2015 [27]), ii) emergence in Manitoba, eastern Ontario, southern Quebec and the Maritimes has been coincident in the recent decades (although with more patchy geographic occurrence in the Maritimes [15, 16]), iii) incidence has been highest in Nova Scotia, but it is lower in Quebec than in Manitoba [9], and iv) the level of background seropositivity not associated with active clinical infections appears to be extremely low [28]. Geographic variations in occurrence of other serologically cross-reacting infections could also explain our observations but we have no evidence of such infections in Canada.

The proportion of samples testing positive overall, and the proportion of individual bands that were positive, varied amongst years, but without a consistent trend so it is difficult to attribute interannual differences to differences over time in sample collection and submission practices. Strain variation remains a possible explanation for interannual variations because evidence of skewed strain frequencies has been found that may be associated with founder events and other processes of the invasion of Canadian habitats by *B*. *burgdorferi* [29–31]. Skewing of strain frequencies by these processes would be expected to be dynamic over time and could perhaps explain the observed interannual variations [31].

The specimens used here had no information on clinical manifestations so it is not possible to know the reason for testing and clinical stage of infection which would be necessary to estimate rates of true and false positivity for C6 and WB results, and patient travel history is unknown. Therefore further investigation of the hypotheses raised here will require prospective studies using panels of samples obtained from patients and controls, a true gold standard for a Lyme disease case (by definitive laboratory tests such as bacterial culture [2] or clinical and laboratory data combined), and contemporaneously collected strain-typed *B*. *burgdorferi* isolates from humans, ticks and reservoir hosts.

In conclusion, we could not find evidence to support simplification of the two-tier algorithm by selecting protein from those used for test interpretation in the current WB algorithm. There were geographic and interannual variations in the proportions of samples reactive to individual proteins in the current WB algorithm that may be consistent with spatio-temporal variations in occurrence of different strains of *B*. *burgdorferi* impacting the WB test result. Further studies are needed to test this hypothesis. Such geographic and temporal variations in responses also reinforce the need to use multiple proteins of the WB in interpretation of Lyme disease test results in Canada.

## Supporting information

S1 File Data used in the study. These data comprise C6 ELISA-positive sera submitted for diagnostic testing to the National Microbiology Laboratory, Public Health Agency of Canada from 2011–2015, and data fields include the overall Western blot result according to US CDC guidelines [3], as well as the results of individual protein bands, the province of origin and the year of submission.
(XLSX)

S2 File Results of statistical analyses. This file comprises tables of the concordance of results of individual proteins of the CDC Western blot algorithm with the overall IgG Western blot, as well as tables of results of logistic regression models.
(DOCX)
