# Supplementary material for: Antibody responses to Borrelia burgdorferi detected by western blot vary geographically in Canada
Source: PLoS One. 2017 Feb 9;12(2):e0171731. doi: 10.1371/journal.pone.0171731 (PMC5300191; doi:10.1371/journal.pone.0171731)
Supplement: S2 File — This file comprises tables of the concordance of results of individual proteins of the CDC Western blot algorithm with the overall IgG Western blot, as well as tables of results of logistic regression models. (DOCX) [file pone.0171731.s002.docx]

S2 File. Results of statistical analyses.

Table A. The concordance of results of individual proteins of the CDC Western blot algorithm with the overall IgG Western blot result as measured by the kappa statistic and relative sensitivity and relative specificity.

| Protein | Relative sensitivity (95% CI) | Relative specificity (95% CI) | Kappa (95% CI) |
| --- | --- | --- | --- |
| p18/21 | 0.689 (0.650-0.729) | 0.964 (0.955-0.972) | 0.711 (0.661-0.739) |
| p25 | 0.640 (0.599-0.681) | 0.916 (0.902-0.927) | 0.570 (0.530-0.608) |
| p28 | 0.147 (0.119-0.179) | 0.990 (0.984-0.994) | 0.194 (0.168-0.219) |
| p30 | 0.397 (0.356-0.439) | 0.893 (0.878-0.906) | 0.315 (0.276-0.354) |
| p39 | 0.889 (0.860-0.913) | 0.892 (0.877-0.905) | 0.709 (0.669-0.748) |
| p41 | 0.996 (0.987-0.999) | 0.098 (0.085-0.111) | 0.044 (0.032-0.056) |
| p45 | 0.652 (0.610-0.691) | 0.955 (0.945-0.963) | 0.648 (0.609-0.687) |
| p58 | 0.528 (0.485-0.570) | 0.881 (0.866-0.895) | 0.414 (0.375-0.453) |
| p66 | 0.788 (0.754-0.823) | 0.722 (0.702-0.742) | 0.400 (0.365-0.435) |
| p93/83 | 0.693 (0.653-0.730) | 0.897 (0.883-0.910) | 0.579 (0.540-0.618) |

Abbreviations: CI = confidence interval

Table B. The parameter values of the logistic regression model in which the IgG Western blot result was the outcome when using data from all provinces.

| Variable | Odds ratio* | 95% confidence interval | Wald z | P |
| --- | --- | --- | --- | --- |
| NS | Reference |  |  |  |
| NFL+PEI+NB | 0.09^a^ | 0.06 – 0.16 | -8.90 | < 0.001 |
| QC+ON | 0.37^b^ | 0.29 – 0.48 | -7.79 | < 0.001 |
| MB | 0.14^c^ | 0.11 – 0.19 | -13.24 | < 0.001 |
| SK+AB+BC | 0.06^d^ | 0.04 – 0.09 | -12.89 | < 0.001 |
| 2011 | Reference |  |  |  |
| 2012 | 0.48^a^ | 0.33 – 0.71 | -3.72 | < 0.001 |
| 2013 | 0.68^b^ | 0.49 – 0.95 | -2.27 | < 0.05 |
| 2014 | 0.49^c^ | 0.35 – 0.69 | -4.09 | < 0.001 |
| 2015 | 0.44^d^ | 0.25 – 0.78 | -2.78 | < 0.01 |

* The odds of being positive are not significantly different amongst province groups that have the same superscripted letters.

Abbreviations: NS = Nova Scotia, NFL = Newfoundland & Labrador, PEI = Prince Edward Island, NB = New Brunswick, QC = Quebec, ON = Ontario, MB = Manitoba, SK = Saskatchewan, AB = Alberta, BC = British Columbia

Table C. The parameter values of the logistic regression model in which the IgG Western blot result was the outcome when using only data from provinces with known *I. scapularis* tick populations.

| Variable | Odds ratio* | 95% confidence interval | Wald z | P |
| --- | --- | --- | --- | --- |
| NS | Reference |  |  |  |
| NB | 0.10^a^ | 0.06 – 0.17 | -8.49 | < 0.001 |
| QC+ON | 0.36^b^ | 0.28 – 0.47 | -7.87 | < 0.001 |
| MB | 0.14^c^ | 0.10 – 0.18 | -13.29 | < 0.001 |
| 2011 | Reference |  |  |  |
| 2012 | 0.51^a^ | 0.34 – 0.75 | -3.36 | < 0.01 |
| 2013 | 0.76^b^ | 0.54 – 1.06 | -1.60 | > 0.05 |
| 2014 | 0.55^c^ | 0.39 – 0.78 | -3.36 | < 0.01 |
| 2015 | 0.58^b^ | 0.32 – 1.05 | -1.79 | > 0.05 |

* The odds of being positive are not significantly different amongst province groups that have the same superscripted letters.

Abbreviations: NS = Nova Scotia, NB = New Brunswick, QC = Quebec, ON = Ontario, MB = Manitoba

Table D. The parameter values of the logistic regression model in which the IgG Western blot result was the outcome when using only data from provinces with known *I. scapularis* tick populations and excluding data from Ontario.

| Variable | Odds ratio* | 95% confidence interval | Wald z | P |
| --- | --- | --- | --- | --- |
| NS | Reference |  |  |  |
| NB | 0.10^a^ | 0.06 – 0.17 | -8.49 | < 0.001 |
| QC | 0.37^b^ | 0.29 – 0.48 | -7.68 | < 0.001 |
| MB | 0.14^c^ | 0.10 – 0.18 | -13.29 | < 0.001 |
| 2011 | Reference |  |  |  |
| 2012 | 0.52^a^ | 0.35 – 0.78 | -3.19 | < 0.01 |
| 2013 | 0.75^b^ | 0.53 – 1.06 | -1.62 | > 0.05 |
| 2014 | 0.55^c^ | 0.38 – 0.78 | -3.34 | < 0.01 |
| 2015 | 0.58^b^ | 0.32 – 1.04 | -1.81 | > 0.05 |

* The odds of being positive are not significantly different amongst province groups that have the same superscripted letters.

Abbreviations: NS = Nova Scotia, NB = New Brunswick, QC = Quebec, MB = Manitoba.

Table E. The parameter values of the logistic regression models in which responses to the individual proteins comprising the IgG Western blot algorithm, were the outcomes when using data from all provinces.

| Variable | Odds ratio* | 95% confidence interval | Wald z | P |
| --- | --- | --- | --- | --- |
|  |  | p18/21 |  |  |
| NS | Reference |  |  |  |
| NFL+PEI+NB | 0.69^a^ | 0.36 –1.32 | -1.12 | > 0.1 |
| QC+ON | 0.69^a^ | 0.48 – 1.00 | -1.94 | 0.05 |
| MB | 0.51^b^ | 0.33 – 0.78 | -3.12 | < 0.01 |
| SK+AB+BC | 0.42^c^ | 0.24 – 0.74 | -3.03 | < 0.01 |
| 2011 | Reference |  |  |  |
| 2012 | 0.44^a^ | 0.26 – 0.76 | -2.99 | < 0.01 |
| 2013 | 0.47^b^ | 0.29 – 0.74 | -3.22 | < 0.01 |
| 2014 | 0.25^c^ | 0.15 – 0.41 | -5.61 | < 0.01 |
| 2015 | 0.47^d^ | 0.22 – 1.02 | -1.91 | > 0.05 |
|  |  | p25 |  |  |
| NS | Reference |  |  |  |
| NFL+PEI+NB | 0.65^a^ | 0.39 –1.09 | -1.64 | > 0.1 |
| QC+ON | 0.87^a^ | 0.64 – 1.19 | -0.87 | > 0.1 |
| MB | 0.45^b^ | 0.32 – 0.64 | -4.48 | < 0.001 |
| SK+AB+BC | 0.20^c^ | 0.12 – 0.32 | -6.35 | < 0.001 |
| 2011 | Reference |  |  |  |
| 2012 | 0.66^a^ | 0.42 – 1.03 | -1.82 | > 0.05 |
| 2013 | 1.28^b^ | 0.87 – 1.87 | -1.26 | > 0.1 |
| 2014 | 0.62^a^ | 0.42 – 0.93 | -2.34 | < 0.05 |
| 2015 | 0.35^c^ | 0.17 – 0.74 | -2.76 | < 0.01 |
|  |  | p28 |  |  |
| NS | Reference |  |  |  |
| NFL+PEI+NB | 0.59^ab^ | 0.19 –1.80 | -0.93 | > 0.1 |
| QC+ON | 0.76^ab^ | 0.46 – 1.27 | -1.06 | > 0.1 |
| MB | 0.43^a^ | 0.21 – 0.87 | -2.37 | < 0.05 |
| SK+AB+BC | 1.01^b^ | 0.48 – 2.13 | 0.01 | > 0.1 |
| 2011 | Reference |  |  |  |
| 2012 | 1.47^a^ | 0.64 – 3.37 | 0.91 | > 0.1 |
| 2013 | 1.33^a^ | 0.64 – 2.74 | 0.77 | > 0.1 |
| 2014 | 1.84^a^ | 0.88 – 3.84 | 1.62 | > 0.1 |
| 2015 | 4.99^b^ | 1.93 – 12.87 | 3.32 | < 0.01 |
|  |  | p30 |  |  |
| NS | Reference |  |  |  |
| NFL+PEI+NB | 1.32^a^ | 0.80 –2.18 | 1.10 | > 0.1 |
| QC+ON | 1.27^a^ | 0.92 – 1.75 | 1.51 | > 0.1 |
| MB | 1.64^b^ | 1.17 – 2.30 | 2.90 | < 0.01 |
| SK+AB+BC | 1.19^a^ | 0.80 – 1.77 | 0.88 | > 0.1 |
|  |  | p39 |  |  |
| NS | Reference |  |  |  |
| NFL+PEI+NB | 0.37^a^ | 0.21 –0.63 | -3.60 | < 0.001 |
| QC+ON | 0.53^b^ | 0.37 – 0.75 | -3.52 | < 0.001 |
| MB | 0.37^c^ | 0.26 – 0.54 | -5.27 | < 0.001 |
| SK+AB+BC | 0.33^d^ | 0.22 – 0.51 | -5.17 | < 0.001 |
|  |  | p41 |  |  |
| NS | Reference |  |  |  |
| NFL+PEI+NB | 0.34^a^ | 0.15 –0.78 | -2.55 | < 0.05 |
| QC+ON | 0.74^b^ | 0.34 – 1.60 | -0.76 | > 0.1 |
| MB | 0.33^c^ | 0.16 – 0.68 | -3.00 | < 0.01 |
| SK+AB+BC | 0.37^d^ | 0.18 – 0.78 | -2.63 | < 0.01 |
| 2011 | Reference |  |  |  |
| 2012 | 1.01^a^ | 0.43 – 2.37 | 0.03 | > 0.1 |
| 2013 | 1.40^a^ | 0.60 – 3.26 | 0.79 | > 0.1 |
| 2014 | 0.28^b^ | 0.13 – 0.59 | -3.34 | < 0.01 |
| 2015 | 0.20^c^ | 0.09 – 0.46 | -3.79 | < 0.001 |
|  |  | p45 |  |  |
| NS | Reference |  |  |  |
| NFL+PEI+NB | 0.68^a^ | 0.36 – 1.27 | -1.22 | > 0.1 |
| QC+ON | 1.04^a^ | 0.74 – 1.48 | 0.27 | > 0.1 |
| MB | 0.43^b^ | 0.29 – 0.65 | -4.07 | < 0.001 |
| SK+AB+BC | 0.40^c^ | 0.24 – 0.68 | -3.40 | < 0.01 |
| 2011 | Reference |  |  |  |
| 2012 | 0.46^a^ | 0.28 – 0.77 | -2.94 | < 0.01 |
| 2013 | 0.83^b^ | 0.54 – 1.27 | -0.87 | > 0.1 |
| 2014 | 0.76^b^ | 0.49 – 1.18 | -1.22 | > 0.1 |
| 2015 | 0.44^b^ | 0.20 – 0.96 | -2.06 | < 0.05 |
|  |  | p66 |  |  |
| NS | Reference |  |  |  |
| NFL+PEI+NB | 1.26^a^ | 0.86 – 1.87 | 1.19 | > 0.1 |
| QC+ON | 1.08^b^ | 0.81 – 1.44 | 0.55 | > 0.1 |
| MB | 1.08^b^ | 0.81 – 1.45 | 0.52 | > 0.1 |
| SK+AB+BC | 1.46^c^ | 1.07 – 1.99 | 2.36 | < 0.05 |
| 2011 | Reference |  |  |  |
| 2012 | 0.90^a^ | 0.64 – 1.27 | -0.61 | > 0.1 |
| 2013 | 0.67^b^ | 0.49 – 0.93 | -2.39 | < 0.05 |
| 2014 | 0.46^b^ | 0.33 – 0.64 | -4.67 | < 0.001 |
| 2015 | 0.38^b^ | 0.24 – 0.61 | -3.95 | < 0.001 |
|  |  | P93/83 |  |  |
| 2011 | Reference |  |  |  |
| 2012 | 1.97^a^ | 1.27 – 3.07 | 3.01 | < 0.01 |
| 2013 | 2.69^b^ | 1.80 – 4.02 | 4.85 | < 0.001 |
| 2014 | 0.73^c^ | 0.48 – 1.11 | -1.47 | > 0.1 |
| 2015 | 0.94^c^ | 0.49 – 1.78 | -0.20 | > 0.1 |

In each model the IgG Western blot result was accounted for and was significant (P < 0.001) but not shown. * For each model, the odds of being positive are not significantly different amongst province groups that have the same superscripted letters. Abbreviations: NS = Nova Scotia, NFL = Newfoundland & Labrador, PEI = Prince Edward Island, NB = New Brunswick, QC = Quebec, ON = Ontario, MB = Manitoba, SK = Saskatchewan, AB = Alberta, BC = British Columbia

Table F. The parameter values of the logistic regression models in which responses to the individual proteins comprising the IgG Western blot algorithm were the outcomes when using only data from provinces with known *I. scapularis* tick populations.

| Variable | Odds ratio* | 95% confidence interval | Wald z | P |
| --- | --- | --- | --- | --- |
|  |  | p18/21 |  |  |
| NS | Reference |  |  |  |
| NB | 0.71^a^ | 0.37 –1.38 | -1.01 | > 0.1 |
| QC+ON | 0.69^b^ | 0.47 – 0.99 | -1.99 | < 0.05 |
| MB | 0.50^c^ | 0.33 – 0.77 | -3.20 | < 0.01 |
| 2011 | Reference |  |  |  |
| 2012 | 0.43^a^ | 0.25 – 0.75 | -2.96 | < 0.01 |
| 2013 | 0.45^b^ | 0.28 – 0.73 | -3.24 | < 0.01 |
| 2014 | 0.25^c^ | 0.15 – 0.42 | -5.36 | < 0.001 |
| 2015 | 0.57^d^ | 0.25 – 1.31 | -1.32 | > 0.05 |
|  |  | p25 |  |  |
| NS | Reference |  |  |  |
| NB | 0.62^a^ | 0.37 –1.05 | -1.78 | > 0.05 |
| QC+ON | 0.86^a^ | 0.63 – 1.17 | -0.95 | > 0.1 |
| MB | 0.44^b^ | 0.31 – 0.63 | -4.61 | < 0.001 |
| 2011 | Reference |  |  |  |
| 2012 | 0.75^ac^ | 0.47 – 1.18 | -1.25 | > 0.1 |
| 2013 | 1.31^b^ | 0.88 – 1.94 | 1.35 | > 0.1 |
| 2014 | 0.67^a^ | 0.45 – 1.01 | -1.89 | > 0.05 |
| 2015 | 0.42^c^ | 0.20 – 0.89 | -2.27 | < 0.05 |
|  |  | p28 |  |  |
| NS | Reference |  |  |  |
| NB | 0.62^a^ | 0.20 –1.94 | -0.82 | > 0.1 |
| QC+ON | 0.76^a^ | 0.46 – 1.27 | -1.03 | > 0.1 |
| MB | 0.44^a^ | 0.21 – 0.90 | -2.24 | < 0.05 |
| 2011 | Reference |  |  |  |
| 2012 | 1.28^a^ | 0.52 – 3.19 | 0.54 | > 0.1 |
| 2013 | 1.34^a^ | 0.63 – 2.87 | 0.76 | > 0.1 |
| 2014 | 1.98^a^ | 0.91 – 4.29 | 1.73 | > 0.05 |
| 2015 | 6.31^b^ | 2.27 – 17.60 | 3.52 | < 0.001 |
|  |  | p30 |  |  |
| NS | Reference |  |  |  |
| NB | 1.29^a^ | 0.77 –2.16 | 0.98 | > 0.1 |
| QC+ON | 1.26^a^ | 0.92– 1.74 | 1.45 | > 0.1 |
| MB | 1.61^b^ | 1.15 – 2.26 | 2.78 | < 0.01 |
|  |  | p39 |  |  |
| NS | Reference |  |  |  |
| NB | 0.31^a^ | 0.17 –0.56 | -3.90 | < 0.001 |
| QC+ON | 0.53^b^ | 0.37 – 0.75 | -3.51 | < 0.001 |
| MB | 0.37^c^ | 0.26 – 0.54 | -5.25 | < 0.001 |
|  |  | p41 |  |  |
| NS | Reference |  |  |  |
| NB | 0.37^a^ | 0.16 –0.86 | -2.31 | < 0.05 |
| QC+ON | 0.75^b^ | 0.35 – 1.64 | -0.71 | > 0.1 |
| MB | 0.34^c^ | 0.16 – 0.69 | -2.95 | < 0.01 |
| 2011 | Reference |  |  |  |
| 2012 | 1.31^a^ | 0.53 – 3.26 | 0.58 | > 0.1 |
| 2013 | 2.02^a^ | 0.81 – 5.10 | 1.50 | > 0.1 |
| 2014 | 0.30^b^ | 0.14 – 0.65 | -3.07 | < 0.01 |
| 2015 | 0.28^c^ | 0.11 – 0.69 | -2.75 | < 0.01 |
|  |  | p45 |  |  |
| NS | Reference |  |  |  |
| NB | 0.60^a^ | 0.31 – 1.15 | -1.55 | > 0.1 |
| QC+ON | 1.04^a^ | 0.74 – 1.47 | 0.22 | > 0.1 |
| MB | 0.43^b^ | 0.29 – 0.64 | -4.12 | < 0.001 |
| 2011 | Reference |  |  |  |
| 2012 | 0.49^a^ | 0.29 – 0.83 | -2.62 | < 0.01 |
| 2013 | 0.85^b^ | 0.55 – 1.33 | -0.71 | > 0.1 |
| 2014 | 0.77^b^ | 0.48 – 1.22 | -1.11 | > 0.1 |
| 2015 | 0.53^b^ | 0.24 – 1.20 | -1.52 | > 0.1 |
|  |  | P58 |  |  |
| 2011 | Reference |  |  |  |
| 2012 | 1.51^a^ | 0.99 – 2.29 | 1.91 | >0.05 |
| 2013 | 0.86^b^ | 0.59 – 1.26 | -0.76 | > 0.1 |
| 2014 | 0.90^b^ | 0.61 – 1.32 | -0.53 | > 0.1 |
| 2015 | 1.85^a^ | 1.03 – 3.31 | -2.07 | < 0.05 |
|  |  | p66 |  |  |
| 2011 | Reference |  |  |  |
| 2012 | 0.94^a^ | 0.65 – 1.36 | -0.34 | >0.1 |
| 2013 | 0.66^b^ | 0.47 – 0.94 | -2.34 | < 0.05 |
| 2014 | 0.46^b^ | 0.33 – 0.65 | -4.41 | < 0.001 |
| 2015 | 0.36^a^ | 0.20 – 0.64 | -3.49 | < 0.001 |
|  |  | P93/83 |  |  |
| 2011 | Reference |  |  |  |
| 2012 | 1.93^a^ | 1.20 – 3.08 | 2.74 | < 0.01 |
| 2013 | 2.46^b^ | 1.62 – 3.73 | 4.22 | < 0.001 |
| 2014 | 0.72^c^ | 0.47 – 1.12 | -1.46 | > 0.1 |
| 2015 | 1.22^c^ | 0.61 – 2.46 | -0.56 | > 0.1 |

In each model the IgG Western blot result was accounted for and was significant (P < 0.001) but not shown. * For each model, The odds of being positive are not significantly different amongst province groups that have the same superscripted letter. Abbreviations: NS = Nova Scotia, NB = New Brunswick, QC = Quebec, ON = Ontario, MB = Manitoba.

Table G. The parameter values of the logistic regression models in which responses to the individual proteins comprising the IgG Western blot algorithm were the outcomes when using only data from provinces with known *I. scapularis* tick populations and excluding data from Ontario.

| Variable | Odds ratio* | 95% confidence interval | Wald z | P |
| --- | --- | --- | --- | --- |
|  |  | p18/21 |  |  |
| NS | Reference |  |  |  |
| NB | 0.71^a^ | 0.37 –1.38 | -1.00 | > 0.1 |
| QC | 0.68^a^ | 0.47 – 0.98 | -2.05 | < 0.05 |
| MB | 0.50^b^ | 0.33 – 0.76 | -3.21 | < 0.01 |
| 2011 | Reference |  |  |  |
| 2012 | 0.45^a^ | 0.26 – 0.80 | -2.73 | < 0.01 |
| 2013 | 0.47^b^ | 0.29 – 0.77 | -3.01 | < 0.01 |
| 2014 | 0.26^c^ | 0.16 – 0.44 | -5.11 | < 0.001 |
| 2015 | 0.60^d^ | 0.26 – 1.37 | -1.21 | > 0.1 |
|  |  | p25 |  |  |
| NS | Reference |  |  |  |
| NB | 0.62^a^ | 0.37 –1.05 | -1.77 | > 0.05 |
| QC | 0.91^a^ | 0.67 – 1.24 | -0.61 | > 0.1 |
| MB | 0.45^b^ | 0.32 – 0.63 | -4.56 | < 0.001 |
| 2011 | Reference |  |  |  |
| 2012 | 0.70^a^ | 0.44 – 1.11 | -1.53 | > 0.1 |
| 2013 | 1.19^b^ | 0.80 – 1.78 | 0.87 | > 0.1 |
| 2014 | 0.61^c^ | 0.40 – 0.93 | -2.31 | < 0.05 |
| 2015 | 0.38^d^ | 0.18 – 0.81 | -2.50 | < 0.05 |
|  |  | p28 |  |  |
| NS | Reference |  |  |  |
| NB | 0.61^a^ | 0.20–1.92 | -0.84 | > 0.1 |
| QC | 0.74^a^ | 0.44 – 1.24 | -1.15 | > 0.1 |
| MB | 0.43^a^ | 0.21 – 0.88 | -2.27 | < 0.05 |
| 2011 | Reference |  |  |  |
| 2012 | 1.40^ac^ | 0.55 – 3.56 | 0.71 | > 0.1 |
| 2013 | 1.46^b^ | 0.66 – 3.22 | 0.94 | > 0.1 |
| 2014 | 2.16^a^ | 0.97 – 4.82 | 1.88 | > 0.05 |
| 2015 | 6.86^c^ | 2.41 – 19.54 | 3.61 | < 0.001 |
|  |  | p30 |  |  |
| NS | Reference |  |  |  |
| NB | 1.30^a^ | 0.77 –2.17 | 0.99 | > 0.1 |
| QC | 1.29^b^ | 0.94– 1.78 | 1.58 | > 0.1 |
| MB | 1.62^b^ | 1.15 – 2.27 | 2.80 | < 0.01 |
|  |  | p39 |  |  |
| NS | Reference |  |  |  |
| NB | 0.31^a^ | 0.17 –0.56 | -3.89 | < 0.001 |
| QC | 0.54^b^ | 0.38 – 0.77 | -3.39 | < 0.01 |
| MB | 0.37^c^ | 0.26 – 0.54 | -5.24 | < 0.001 |
|  |  | p41 |  |  |
| NS | Reference |  |  |  |
| NB | 0.37^a^ | 0.16 –0.86 | -2.31 | < 0.05 |
| QC | 0.74^b^ | 0.34 – 1.61 | -0.76 | > 0.1 |
| MB | 0.34^c^ | 0.16 – 0.69 | -2.95 | < 0.01 |
| 2011 | Reference |  |  |  |
| 2012 | 1.33^a^ | 0.53 – 3.31 | 0.61 | > 0.1 |
| 2013 | 2.09^a^ | 0.83 – 5.27 | 1.57 | > 0.1 |
| 2014 | 0.31^b^ | 0.15 – 0.67 | -2.99 | < 0.01 |
| 2015 | 0.28^c^ | 0.11 – 0.71 | -2.68 | < 0.01 |
|  |  | p45 |  |  |
| NS | Reference |  |  |  |
| NB | 0.60^a^ | 0.31 – 1.15 | -1.54 | > 0.1 |
| QC | 1.04^a^ | 0.74 – 1.48 | 0.25 | > 0.1 |
| MB | 0.43^b^ | 0.29 – 0.64 | -4.12 | < 0.001 |
| 2011 | Reference |  |  |  |
| 2012 | 0.50^a^ | 0.29 – 0.86 | -2.52 | < 0.01 |
| 2013 | 0.86^b^ | 0.54 – 1.35 | -0.67 | > 0.1 |
| 2014 | 0.78^b^ | 0.48 – 1.24 | -1.06 | > 0.1 |
| 2015 | 0.54^b^ | 0.24 – 1.21 | -1.49 | > 0.1 |
|  |  | P58 |  |  |
| 2011 | Reference |  |  |  |
| 2012 | 1.64^a^ | 1.07 – 2.52 | 2.25 | < 0.05 |
| 2013 | 0.93^b^ | 0.63 – 1.38 | -0.35 | > 0.1 |
| 2014 | 0.98^b^ | 0.66 – 1.45 | -0.11 | > 0.1 |
| 2015 | 2.01^a^ | 1.11 – 3.61 | 2.33 | < 0.05 |
|  |  | p66 |  |  |
| 2011 | Reference |  |  |  |
| 2012 | 0.89^a^ | 0.61 – 1.30 | -0.61 | >0.1 |
| 2013 | 0.65^b^ | 0.46 – 0.92 | -2.40 | < 0.05 |
| 2014 | 0.45^b^ | 0.32 – 0.64 | -4.43 | < 0.001 |
| 2015 | 0.35^a^ | 0.20 – 0.63 | -3.53 | < 0.001 |
|  |  | P93/83 |  |  |
| 2011 | Reference |  |  |  |
| 2012 | 1.96^a^ | 1.22 – 3.16 | 2.78 | < 0.01 |
| 2013 | 2.50^b^ | 1.64 – 3.83 | 4.26 | < 0.001 |
| 2014 | 0.74^c^ | 0.48 – 1.15 | -1.34 | > 0.1 |
| 2015 | 1.25^c^ | 0.62 – 2.52 | 0.62 | > 0.1 |

In each model the IgG Western blot result was accounted for and was significant (P < 0.001) but not shown. * For each model, The odds of being positive are not significantly different amongst province groups that have the same superscripted letter. Abbreviations: NS = Nova Scotia, NB = New Brunswick, QC = Quebec, MB = Manitoba.

Table H. The parameter values of the logistic regression models in which responses to the individual proteins comprising the IgG Western blot algorithm were the outcomes when using only data from provinces with known *I. scapularis* tick populations, and using only data from IgG WB-positive samples when these analyses yielded significant inter-province or inter-annual variations.

| Variable | Odds ratio* | 95% confidence interval | Wald z | P |
| --- | --- | --- | --- | --- |
|  |  | p18/21 |  |  |
| NS | Reference |  |  |  |
| NB | 0.66 | 0.35 – 3.64 | 0.19 | > 0.1 |
| QC+ON | 0.66 | 0.43 – 1.01 | -1.90 | > 0.05 |
| MB | 0.53 | 0.31 – 0.90 | -2.34 | < 0.05 |
| 2011 | Reference |  |  |  |
| 2012 | 0.34 | 0.16 – 0.72 | -2.80 | < 0.01 |
| 2013 | 0.41 | 0.22 – 0.79 | -2.67 | < 0.01 |
| 2014 | 0.31 | 0.16 – 0.60 | -3.44 | < 0.01 |
| 2015 | 1.70 | 0.35 – 8.21 | 0.65 | > 0.05 |
|  |  | p25 |  |  |
| 2011 | Reference |  |  |  |
| 2012 | 1.10 | 0.59 – 2.07 | 0.30 | > 0.1 |
| 2013 | 2.19 | 1.29 – 3.72 | 2.91 | < 0.01 |
| 2014 | 1.09 | 0.64 – 1.87 | 0.32 | > 0.1 |
| 2015 | 0.58 | 0.22 – 1.54 | -1.09 | > 0.1 |
|  |  | p28 |  |  |
| NS | Reference |  |  |  |
| NB | 0.38 | 0.08 – 1.84 | -1.20 | > 0.1 |
| QC+ON | 0.66 | 0.38 – 1.15 | -1.48 | > 0.1 |
| MB | 0.42 | 0.19 -0.94 | -2.10 | < 0.05 |
| 2011 | Reference |  |  |  |
| 2012 | 1.42 | 0.56 – 3.58 | 0.75 | > 0.1 |
| 2013 | 1.13 | 0.52 – 2.49 | 0.33 | > 0.1 |
| 2014 | 1.91 | 0.86 – 4.24 | 1.59 | > 0.1 |
| 2015 | 6.20 | 1.98 – 19.43 | 3.13 | < 0.01 |

Abbreviations: NS = Nova Scotia, NB = New Brunswick, QC = Quebec, ON = Ontario, MB = Manitoba.

Table I. The parameter values of the ordered logistic regression model in which the outcome was the number of bands that were positive in IgG Western blot negative samples.

| Variable | Odds ratio* | 95% confidence interval | Wald z | P |
| --- | --- | --- | --- | --- |
| NS | Reference |  |  |  |
| NFL+PEI+NB | 0.46^a^ | 0.32 – 0.66 | -4.24 | < 0.001 |
| QC+ON | 0.74^b^ | 0.56 – 0.99 | -1.99 | < 0.05 |
| MB | 0.45^c^ | 0.34 – 0.59 | -5.52 | < 0.001 |
| SK+AB+BC | 0.49^d^ | 0.36 – 0.66 | -4.76 | < 0.001 |
| 2011 | Reference |  |  |  |
| 2012 | 0.78^a^ | 0.56 – 1.10 | -1.42 | > 0.1 |
| 2013 | 0.89^a^ | 0.65 – 1.23 | -0.68 | > 0.1 |
| 2014 | 0.28^b^ | 0.21 – 0.39 | -7.71 | < 0.001 |
| 2015 | 0.24^c^ | 0.15 – 0.37 | -6.51 | < 0.001 |

* Odds ratios with the same superscripted letter are not significantly different.

Abbreviations: NS = Nova Scotia, NFL = Newfoundland & Labrador, PEI = Prince Edward Island, NB = New Brunswick, QC = Quebec, ON = Ontario, MB = Manitoba, SK = Saskatchewan, AB = Alberta, BC = British Columbia
